# Supplementary figures and images for: Fluconazole worsened lung inflammation, partly through lung microbiome dysbiosis in mice with ovalbumin-induced asthma
Source: PeerJ. 2024 Oct 28;12:e18421. doi: 10.7717/peerj.18421 (PMC11526796; doi:10.7717/peerj.18421)

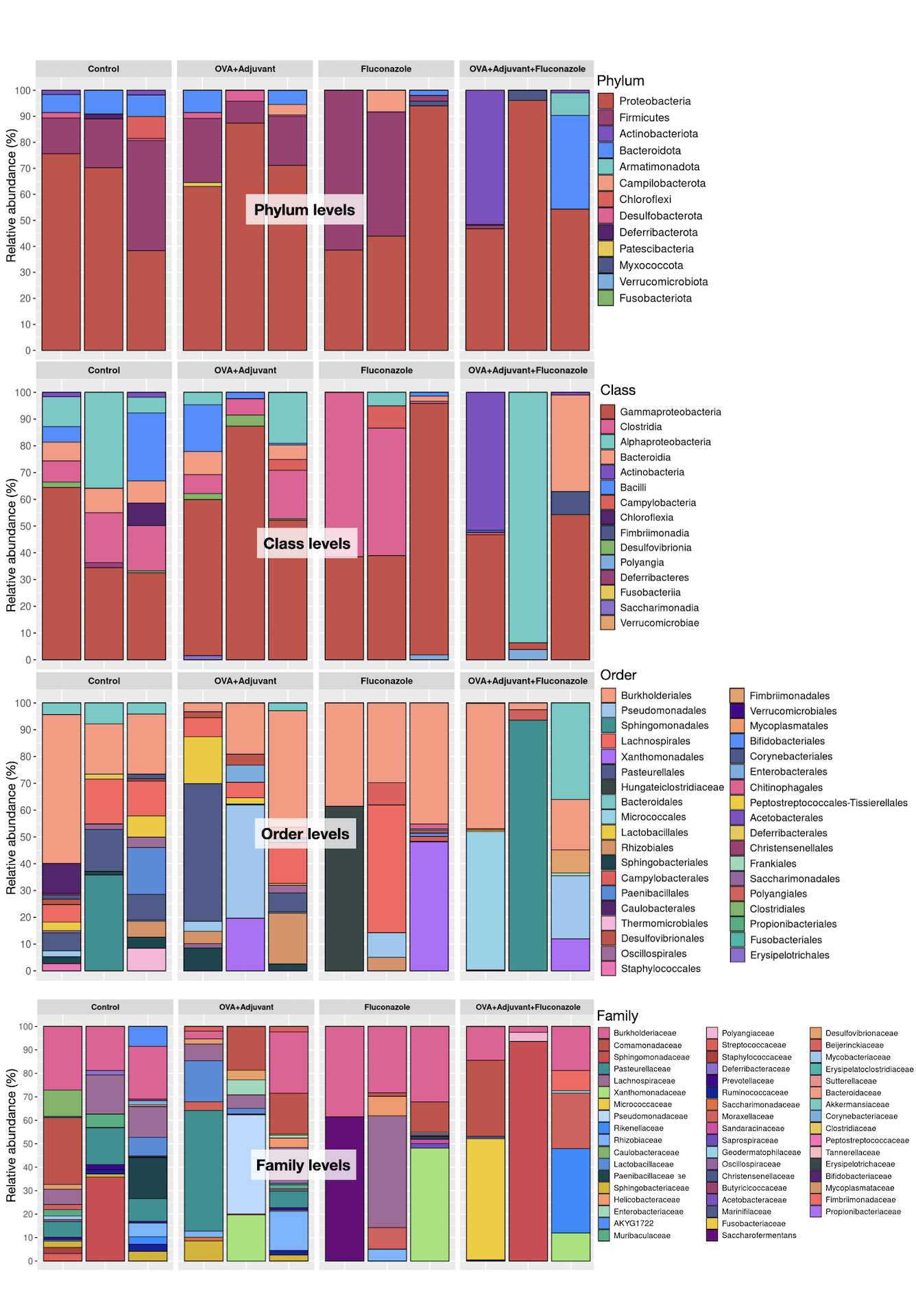

Supplement: Supplemental Information 1 [file peerj-12-18421-s001.png]
