# Supplementary material for: Fluconazole worsened lung inflammation, partly through lung microbiome dysbiosis in mice with ovalbumin-induced asthma
Source: PeerJ. 2024 Oct 28;12:e18421. doi: 10.7717/peerj.18421 (PMC11526796; doi:10.7717/peerj.18421)
Supplement: Supplemental Information 2 [file peerj-12-18421-s002.pdf]

16S rRNA Sequencing Report

Project ID: 23S0013

Sample received date: 5-07-2023

Reported date: 25-09-2023

Sample type: DNA

Sample size: 12 samples

16S rRNA Metagenomic Sequencing Report

☑ Quality profiles
☑ Alpha diversity
☑ Beta diversity
☑ Taxonomic profiles and Krona plots
☑ Linear discriminant analysis effect size (LEfSe) and Cladogram

Optional Bioinformatics

☐ Phylogenetic tree
☑ Heatmap of relative abundance
☐ Venn diagram
☐ Metabolic pathway prediction
☑ NCBI Genbank submission

Reported by ____________________

Lampet Wongsaroj, M.Sc, Ph.D

Scientist

____

Kritsakorn Saninjuk, M.Sc, Ph.D

Scientist

16S rRNA Sequencing

Results

1. Sample information

The sample information table shows your sample ID, sample groups, and the names of fastq files obtained from 16S rRNA Metagenomic Sequencing. In this report, only 10 samples are shown in the following table. If there are more than 10 samples in a project, the complete sample information table can be accessed from the link below the table.

No.	Customer label	Sample ID	Group	Raw seq R1 file (fastq)	Raw seq R2 file (fastq)
1	Control Lung 1	Control-Lung1	Control	Control-Lung1_S10_ L001_R1_001.fastq	Control-Lung1_S10_ L001_R2_001.fastq
2	Control Lung 2	Control-Lung2	Control	Control-Lung2_S11_ L001_R1_001.fastq	Control-Lung2_S11_ L001_R2_001.fastq
3	Control Lung 3	Control-Lung3	Control	Control-Lung3_S12_ L001_R1_001.fastq	Control-Lung3_S12_ L001_R2_001.fastq
4	Fluco Lung 1	Fluco-lung1	Fluconazole	Fluco-lung1_S13_ L001_R1_001.fastq	Fluco-lung1_S13_ L001_R2_001.fastq
5	Fluco Lung 2	Fluco-lung2	Fluconazole	Fluco-lung2_S14_ L001_R1_001.fastq	Fluco-lung2_S14_ L001_R2_001.fastq
6	Fluco Lung 3	Fluco-lung3	Fluconazole	Fluco-lung3_S15_ L001_R1_001.fastq	Fluco-lung3_S15_ L001_R2_001.fastq
7	OVA+Fluco Lung 1	OVA-Fluco-lung1	OVA+Adjuvant+ Fluconazole	OVA-Fluco-lung1_S16_ L001_R1_001.fastq	OVA-Fluco-lung1_S16_ L001_R2_001.fastq
8	OVA+Fluco Lung 2	OVA-Fluco-lung2	OVA+Adjuvant+ Fluconazole	OVA-Fluco-lung2_S17_ L001_R1_001.fastq	OVA-Fluco-lung2_S17_ L001_R2_001.fastq
9	OVA+Fluco Lung 3	OVA-Fluco-lung3	OVA+Adjuvant+ Fluconazole	OVA-Fluco-lung3_S18_ L001_R1_001.fastq	OVA-Fluco-lung3_S18_ L001_R2_001.fastq
10	OVA Lung 1	OVA-Lung1	OVA+Adjuvant	OVA-Lung1_S10_ L001_R1_001.fastq	OVA-Lung1_S10_ L001_R2_001.fastq

Raw seq files:
All FASTQ files
Table:
Summary_samples_information.txt

2. Quality profiles

Track Read Changes

The numbers of sequence read from all samples in each step of the pipeline analysis are shown to estimate the performance of the run and how do reads change through the pipeline. More than 50,000 reads are recommended from raw sequencing reads.

- Trimming is used for removing the primer and adaptor sequences from V3V4 amplicon reads.
- Filtering is used to filter low-quality sequences of both forward and reverse reads, which are unexpected reads. Normally, the quality of reverse read drops off at the end of read more than in the forward read.
- Denoising refers to a process that removes sequence errors from amplicon reads.
- Merging of paired-end reads generates one consensus sequence by assembly between the forward and reverse overlapping reads.
- Nonchim (refers to non-chimeric reads) is a process to remove chimeric reads. Chimeras occur during PCR reaction step when two sequencing reads incorrectly joined together. They are indicative of 16S structural variation.
- Percentage calculates the percent of remaining sequencing reads after non-chimeric process from total reads at the first step.

In this report, only 10 samples are provided in the following table. If there are more than 10 samples in your project, the complete sample information table can be accessed from the link below the table.

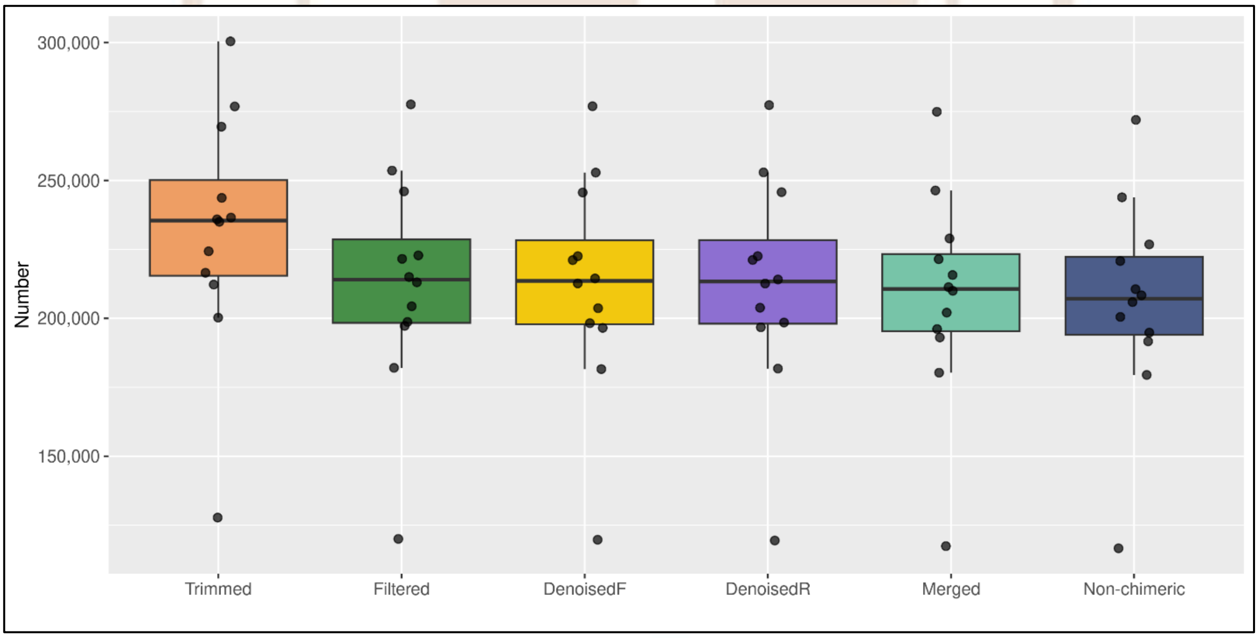

Sample ID	Trimmed-V3V4	Filtered	DenoisedF	DenoisedR	Merged	Nonchim	Remove mitochondria
Control Lung 1	224,328	204,371	203,676	203,847	202,078	200,518	89,127
Control Lung 2	200,258	182,056	181,580	181,780	180,289	179,480	100,919
Control Lung 3	269,503	246,036	245,639	245,763	228,958	226,815	86,703
Fluco Lung 1	212,240	197,270	196,539	196,753	193,069	191,648	64,884
Fluco Lung 2	216,516	198,707	198,253	198,482	196,120	194,822	67,696
Fluco Lung 3	235,897	214,996	214,469	214,104	211,327	210,577	97,788
OVA+Fluco Lung 1	127,737	120,010	119,703	119,429	117,395	116,599	14,492
OVA+Fluco Lung 2	235,004	221,567	221,122	221,171	215,710	205,907	26,521
OVA+Fluco Lung 3	276,844	253,586	252,858	252,927	246,379	243,880	65,220
OVA Lung 1	300,446	277,594	276,922	277,328	274,915	271,981	94,720

Figure:
Box_plot_summary_filter.png
Table:
Summary_filter_reads_rm_mitochondria.txt

PORCINOTEC CO., LTD.

Rarefaction analysis

Rarefaction curve represents the species richness (the number of different species) within and between sequencing reads. It can be used to estimate how many amplicon sequence variants (ASVs) or taxa would have been found in the same size of reads (1). Rarefaction curve rapidly increases at first where every read in the samples are identified (like the exponential phase), then slowly reaches the plateau stage when the rare species remain to be sampled (like the stationary phase). The plateau curve in rarefaction analysis determines whether sufficient reads have been detected to get a good representation of the microbial compositions in an environment. More samples can increase the number of reads with good representatives of all taxa. The figure of rarefaction curve can be downloaded with the same filename below the figure.

Suggestion by Porcinotec:

The approximate saturation of microbial richness of all samples was 14,492 sequencing depths, as estimated by the rarefaction curves. The plateau curve in rarefaction was observed, when approximately 5,000 sequencing depths were reached, excepted Fluco Lung3. This finding sufficiently estimated the true bacterial compositions of microbiome in mice among the sample groups. The observed numbers of ASVs were varied among the samples. Control group showed the highest number of observed ASVs, while OVA+Adjuvant+Fluconazole showed the lowest number of observed ASVs. The data suggested that OVA+Adjuvant+Fluconazole had an effect on the number of observed ASVs compared to control.

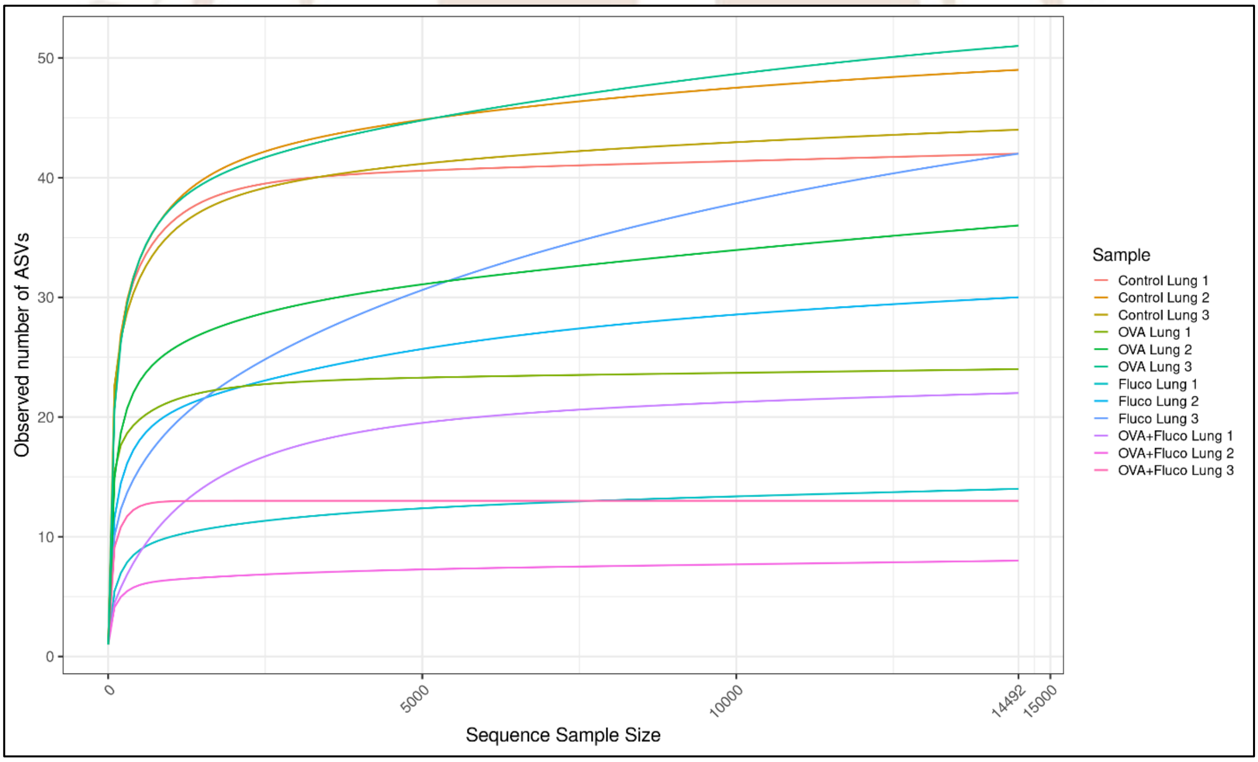

Figure:

Rarefaction_curves.png

3. Alpha diversity

Alpha diversity analyses refer to bacterial diversity within each community (1). Box plots of alpha diversity (observed species, Chao1, Shannon, and phylogenetic diversity (PD) whole tree) in each sample group are shown. The black dots represent individual samples in each group. Alpha diversity values of each sample and quartiles of the distribution (minimum, first quartile, median, third quartile, and maximum of boxes) can be found. The alpha-diversity values are also provided as the table from the link below figure.

Suggestion by Porcinotec:

High quality reads of 16S rRNA after processing were 955,065 reads. The observed abundance of ASVs was the highest in control, followed by OVA+Adjuvant, Fluconazole, and OVA+Adjuvant+Fluconazole, respectively. Moreover, the similar results were observed in Chao1 richness index and Shannon evenness. However, there were no statistically significant difference among the sample groups (Kruskal Wallis test; p>0.05). These findings illustrated that the highest bacterial abundance (Chao1) and diversity (Shannon) were found in control, while the lowest microbiota was observed in OVA+Adjuvant+Fluconazole.

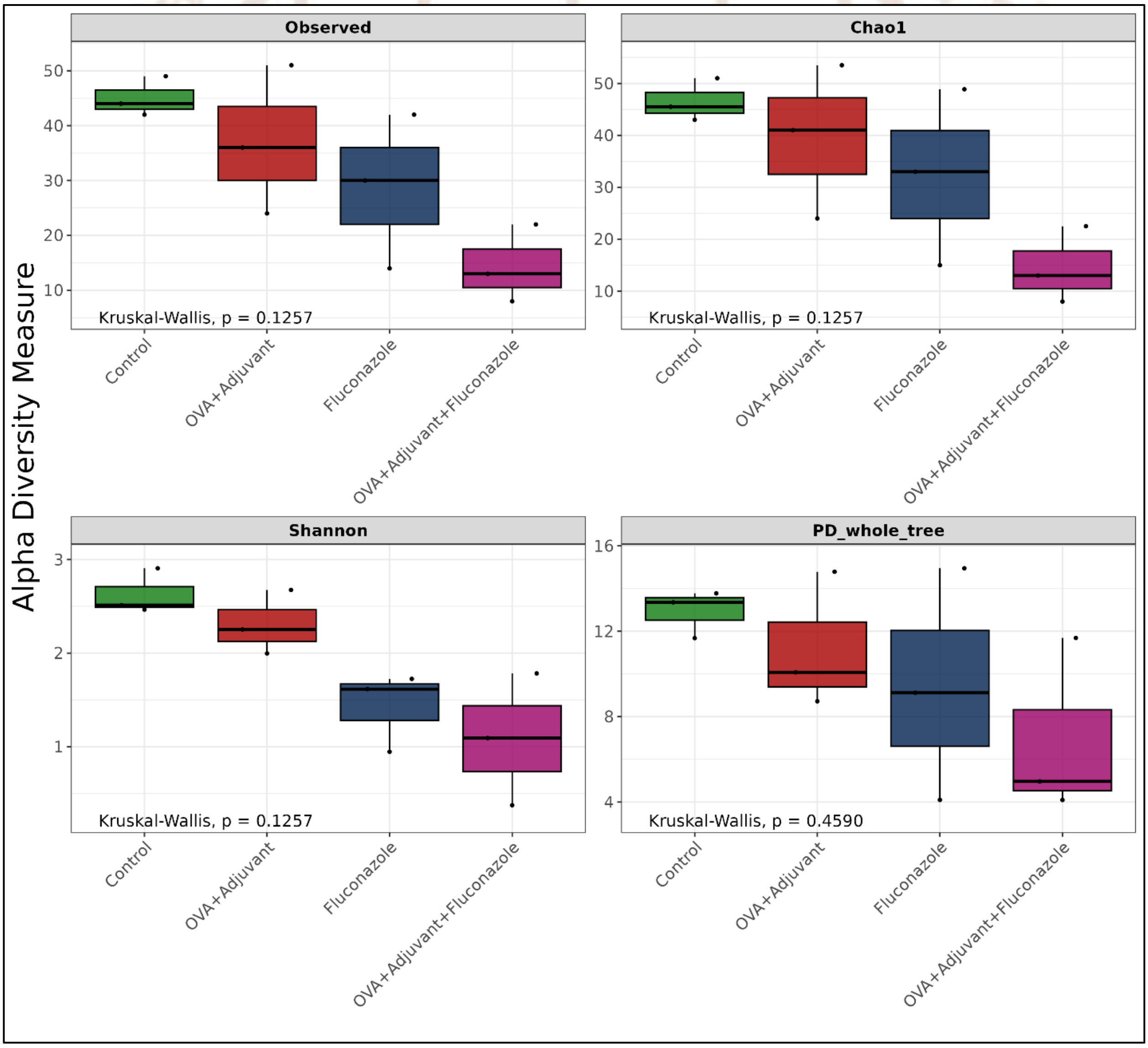

No.	Sample ID	Reads	Observed ASVs	Chao1	Shannon	PD whole tree
1	Control Lung 1	89,127	42	43	2.47	11.68
2	Control Lung 2	100,919	49	51	2.51	13.78
3	Control Lung 3	86,703	44	45.5	2.91	13.35
4	Fluco Lung 1	64,884	14	15	0.95	4.10
5	Fluco Lung 2	67,696	30	33	1.72	9.12
6	Fluco Lung 3	97,788	42	48.87	1.62	14.95
7	OVA+Fluco Lung 1	14,492	22	22.5	1.09	11.69
8	OVA+Fluco Lung 2	26,521	8	8	0.37	4.10
9	OVA+Fluco Lung 3	65,220	13	13	1.78	4.97
10	OVA Lung 1	94,720	24	24	2.25	8.72

Figure:
plot_richness_boxplot_Group.png
HTML:
plot_richness_boxplot_Group.html
Table:
Summary_richness.txt

PORCINOTEC CO., LTD.

4. Beta diversity

Beta diversity analyses represent the difference in microbial community between samples, or a simpler definition would be, how similar or different it is between your samples. To estimate the beta diversity, principal coordinate analysis (PCoA) on weight/unweight Unifrac distances, generalized UniFrac (GUniFrac) distances, and non-metric multidimensional scaling (NMDS) based on Bray-Curtis dissimilarity are usually performed (2).

PCoA is a multidimensional scaling method to visualize the dissimilarity of data (3). It uses the actual distances between samples as input for the dissimilarity matric. UniFrac distances measure the phylogenetic distance between a pair of samples (2). Weighted distance takes into account the relative abundance of taxa shared between samples combined with phylogenetic distance, but unweighted UniFrac distance considers a qualitative diversity metric (only presence/absence of taxa in a sample). However, these are limited to both rare phylogenies for unweighted UniFrac distance and most abundant lineages for weighted UniFrac distances. Therefore, GUniFrac distance has been developed to overcome the limitations on two UniFrac distances (4). Thus, GUniFrac distance can be used to detect a much wider range of changed microbiota composition.

NMDS is non-parametric approach (3). The actual distances between samples are converted into rank orders for creating the dissimilarity matric. The Bray-Curtis dissimilarity is calculated based on non-phylogenetic measurement with microbial abundance between a pair of samples (similar to weighted Unifrac). Please note that NMDS is calculated based on non-metric distances, while PCoA is a matrix of dissimilarities between samples.

	**Parametric distances**	**Non-parametric distances**	**Phylogenetic**	**Relative abundance**
PCoA Weighted UniFrac	✓	-	✓	✓
PCoA Unweighted UniFrac	✓	-	✓	-
NMDS Bray-Curtis	-	✓	-	✓

For beta-diversity plots, the 2-dimensional PCoA on weighted/unweighted Unifrac distances, GUniFrac distances, and NMDS based on Bray-Curtis dissimilarity are generated as a pdf document. In addition, PERMANOVA testing is also provided across all sample groups and pairwise between samples. The customer can propose the parameters of interest to be analyzed.

Suggestion by Porcinotec:

The GUniFrac with alpha 0.5 showed that microbiota communities were clearly separated among the sample groups (PERMANOVA test; p = 0.03796). However, other beta-diversity plots were no significant difference among the groups. Moreover, the boxplot distance metric to control obtained from GUniFrac showed that Fluconazole and OVA+Adjuvant+Fluconazole were statically significant difference from the control (p=0.0029).

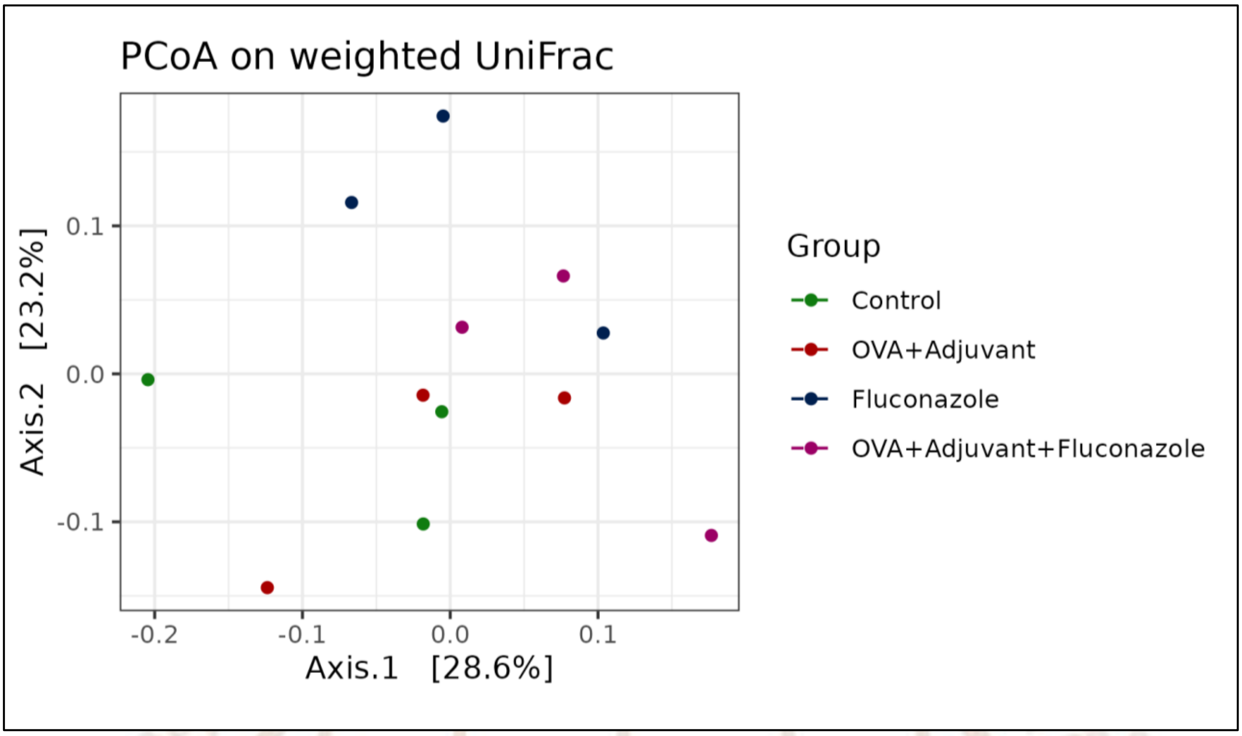
PCoA on weighted UniFrac
Axis.2 [23.2%]
0.1
0.0
-0.1
-0.2
-0.1
0.0
0.1
Axis.1 [28.6%]
Group
Control
OVA+Adjuvant
Fluconazole
OVA+Adjuvant+Fluconazole

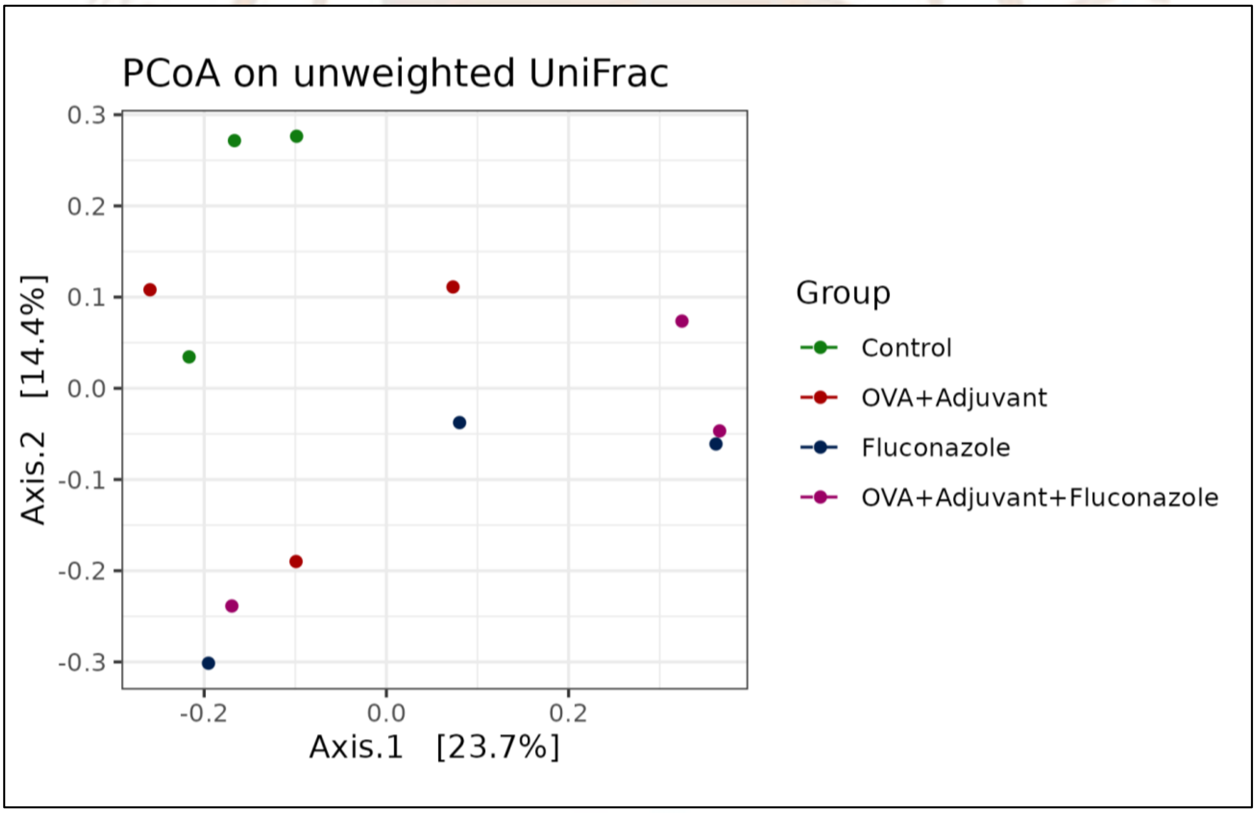
PCoA on unweighted UniFrac
Axis.2 [14.4%]
0.3
0.2
0.1
0.0
-0.1
-0.2
-0.3
-0.2
0.0
0.2
Axis.1 [23.7%]
Group
Control
OVA+Adjuvant
Fluconazole
OVA+Adjuvant+Fluconazole

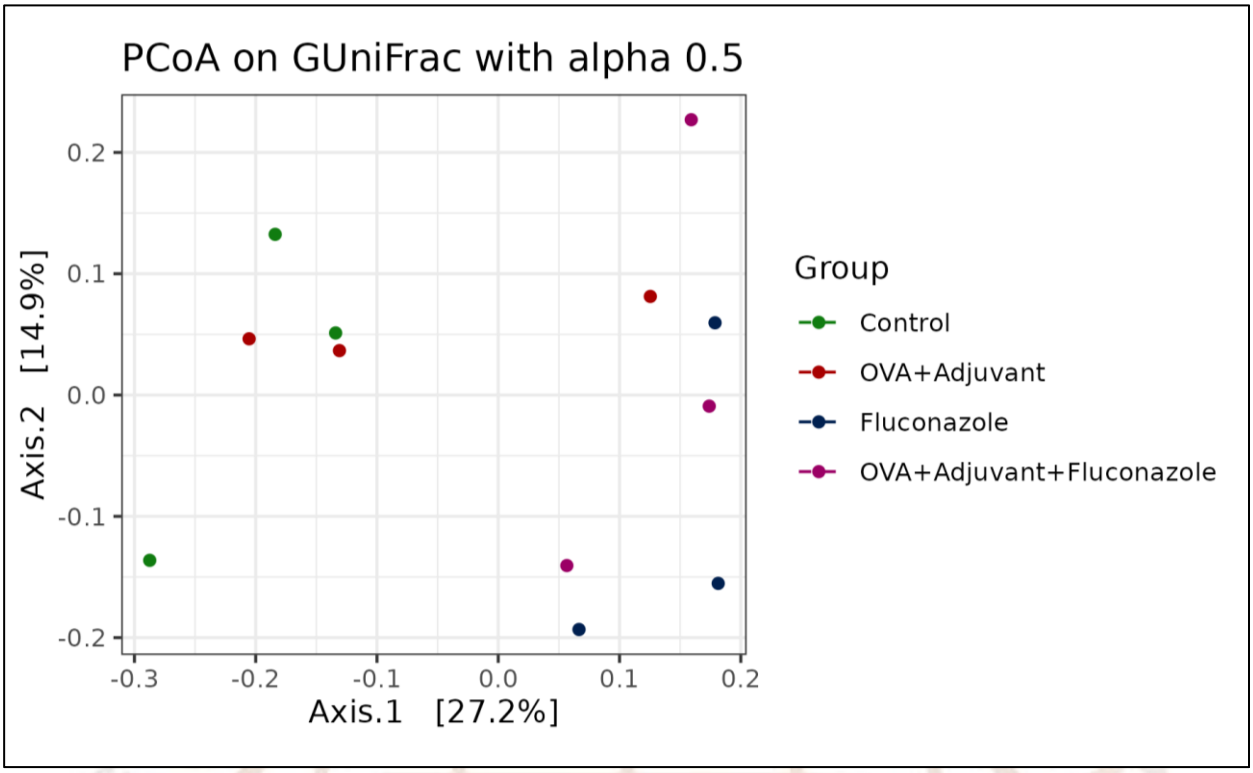
PCoA on GUniFrac with alpha 0.5
Axis.2 [14.9%]
Axis.1 [27.2%]
0.2
0.1
0.0
-0.1
-0.2
-0.3
-0.2
-0.1
0.0
0.1
0.2
Group
Control
OVA+Adjuvant
Fluconazole
OVA+Adjuvant+Fluconazole

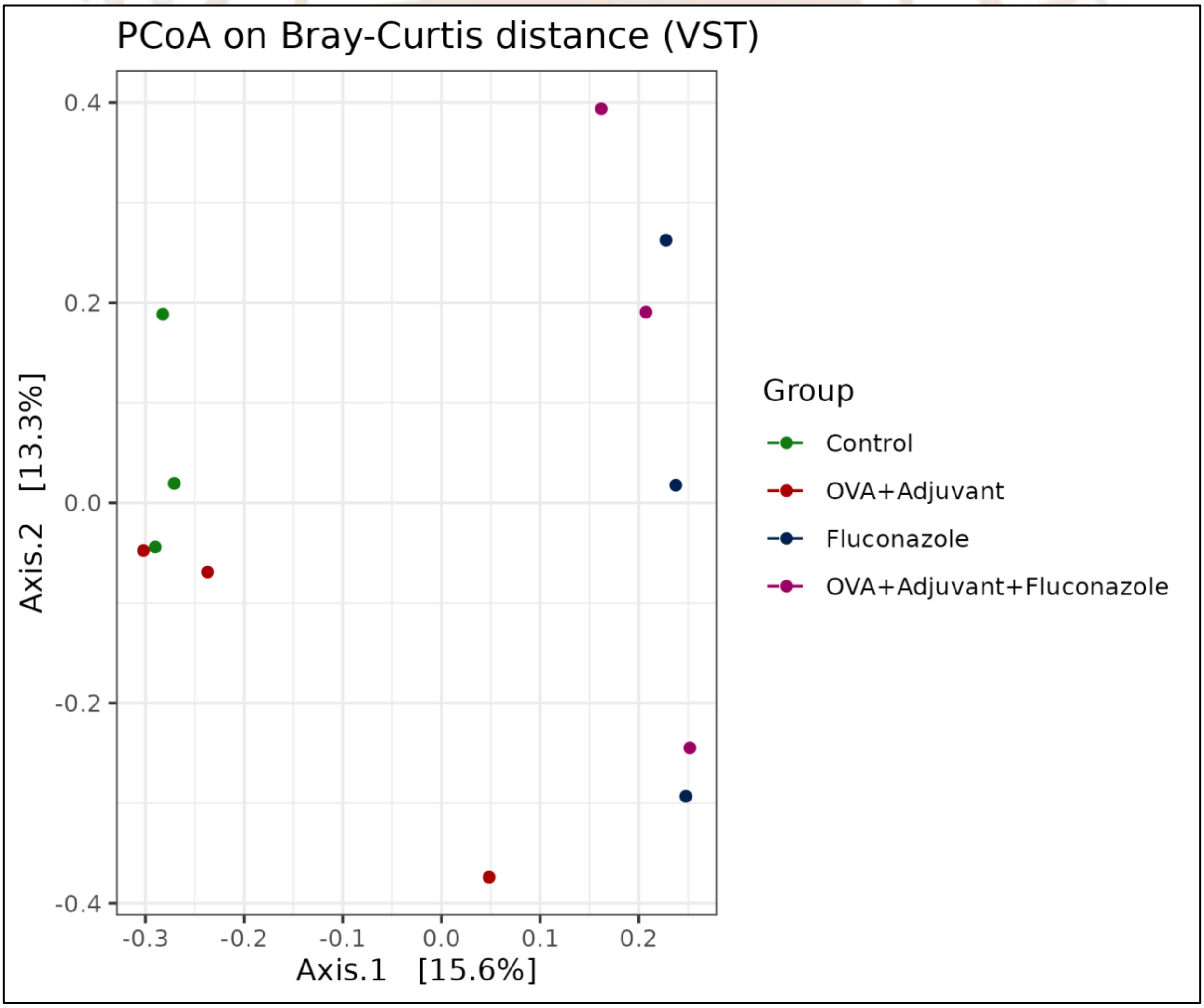
PCoA on Bray-Curtis distance (VST)
Axis.2 [13.3%]
Axis.1 [15.6%]
0.4
0.2
0.0
-0.2
-0.4
-0.3
-0.2
-0.1
0.0
0.1
0.2
Group
Control
OVA+Adjuvant
Fluconazole
OVA+Adjuvant+Fluconazole

PDF:
plot_ordination_Group.pdf
Figure:
plot_ordination_Unweighted_UniFrac_Group.png
plot_ordination_Weighted_UniFrac_Group.png
plot_ordination_GUniFrac_with_alpha_0.5_Group.png
plot_ordination_Bray-Curtis_Group.png
Boxplot_GUniFrac with alpha 0.5_distance.png
HTML:
plot_Beta_diversity_PCoA_on_unweighted_UniFrac.html
plot_Beta_diversity_PCoA_on_weighted_UniFrac.html
plot_Beta_diversity_PCoA_on_GUniFrac_with_alpha_0.05.html
plot_Beta_diversity_PCoA_on_PCoA_Bray_Curtis_distance.html
PERMANOVA Table:
statistic_PERMANOVA_Bray-Curtis_beta_diversity.txt
statistic_PERMANOVA_GUniFrac_with_alpha0.5_beta_diversity.txt
statistic_PERMANOVA_Unweighted_UniFrac_beta_diversity.txt
statistic_PERMANOVA_Weighted_UniFrac_beta_diversity.txt
statistic_PERMANOVA_Distance_GUniFrac with alpha 0.5_beta_diversity.txt

5. Taxonomic profiles

Bar chart

Microbiota compositions of different taxa profiles (phylum, class, order, family, and genus) are frequently visualized by bar charts. Different color represents different taxa compositions. Microbial composition graph of each taxonomy level and abundance tables can be accessed from the link below the figure. You can visualize the abundance of each bacterium on the HTML files.

Suggestion by Porcinotec:

A total of 13 different bacterial phyla were identified. The bacteria of phylum Proteobacteria were highly prevalent (avg. 64.95±6.02%), followed by Firmicutes, Bacteroidota, and Actinobacteriota, respectively. The Proteobacteria was increased in OVA+Adjuvant (avg. 73.84±7.15%) compared to control (avg. 6.41±11.62%). The Firmicutes was increased in Fluconazole (avg. 37.13±17.9%) when compared to the control (avg. 24.95±8.82%), while Bacteroidota was lower in this group. The member of Firmicutes was decreased in OVA+Adjuvant+Fluconazole compared to other 3 groups.

Overall, 77 genera were detected among samples. There was high variation in lung microbiome among the samples. Interestingly, the member of *Burkholderia-Caballeronia-Paraburkholderia* was increased in Fluconazole (avg. 50.29±21.35%) compared to control (avg. 24.15±4.08%), while it was decreased in both OVA+Adjuvant+Fluconazole and OVA+Adjuvant groups. This data indicated that both OVA+Adjuvant and Fluconazole had an effect on *Burkholderia-Caballeronia-Paraburkholderia*. OVA+Adjuvant could decrease the composition of *Burkholderia-Caballeronia-Paraburkholderia*, while Fluconazole might increase the population of this bacterium. However, OVA+Adjuvant had a higher effect on lung microbiome than Fluconazole, which could be observed from the decrease of this bacterium in OVA+Adjuvant+Fluconazole group as well as OVA+Adjuvant.

Phylum

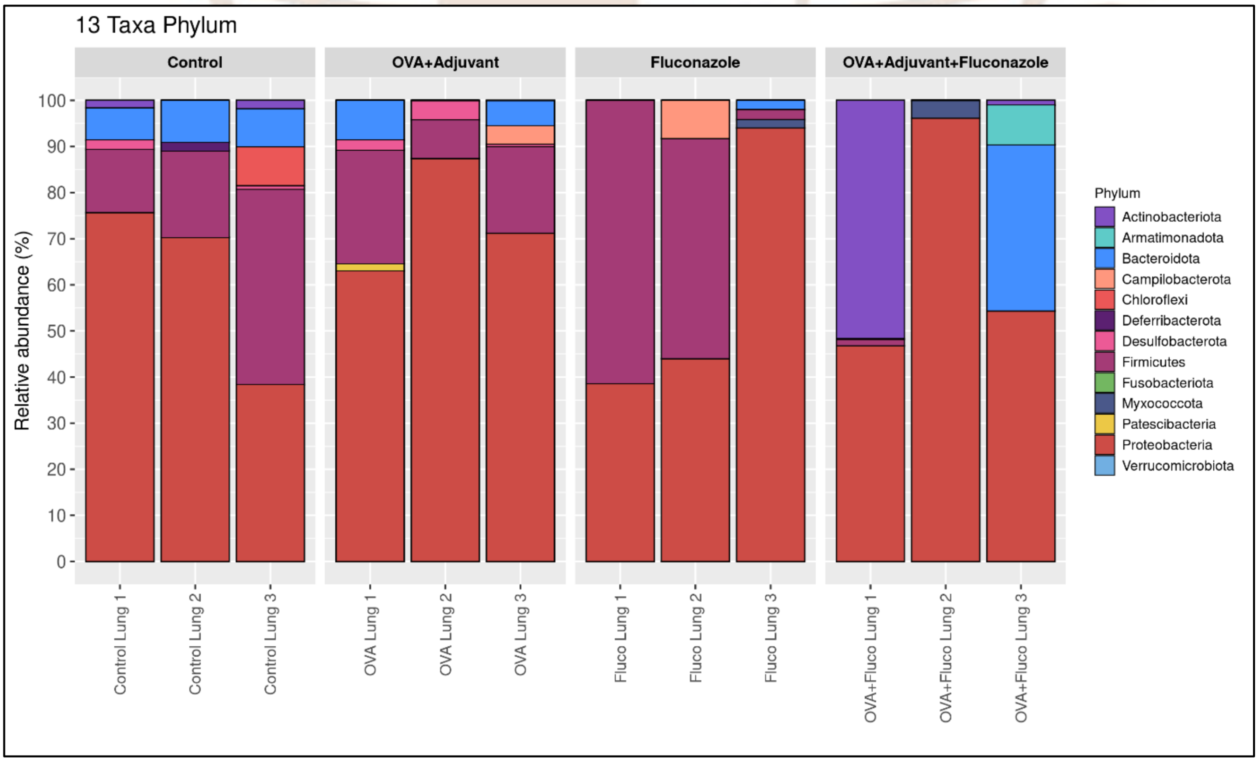

Class

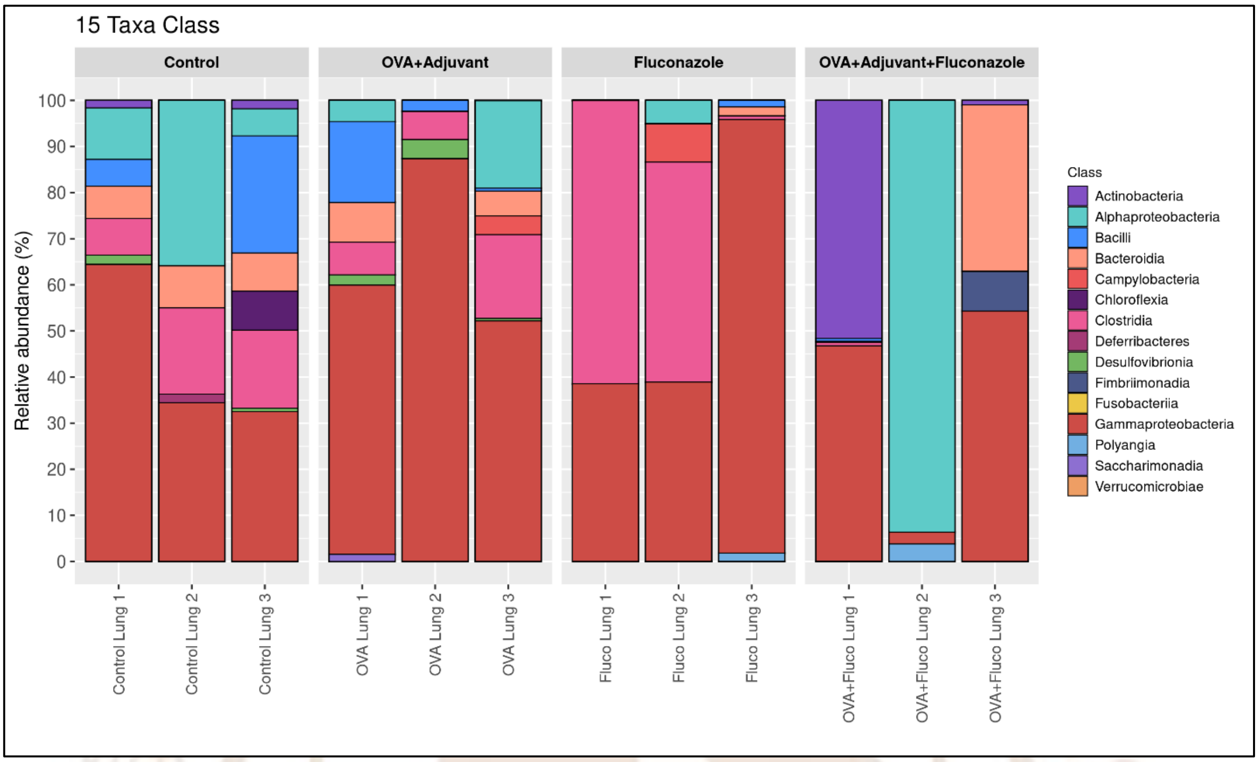
15 Taxa Class
Control
OVA+Adjuvant
Fluconazole
OVA+Adjuvant+Fluconazole
Relative abundance (%)
100
90
80
70
60
50
40
30
20
10
0
Control Lung 1
Control Lung 2
Control Lung 3
OVA Lung 1
OVA Lung 2
OVA Lung 3
Fluco Lung 1
Fluco Lung 2
Fluco Lung 3
OVA+Fluco Lung 1
OVA+Fluco Lung 2
OVA+Fluco Lung 3
Class
Actinobacteria
Alphaproteobacteria
Bacilli
Bacteroidia
Campylobacteria
Chloroflexia
Clostridia
Deferribacteres
Desulfovibrionia
Fimbriimonadia
Fusobacteriia
Gammaproteobacteria
Polyangia
Saccharimonadia
Verrucomicrobiae

Order

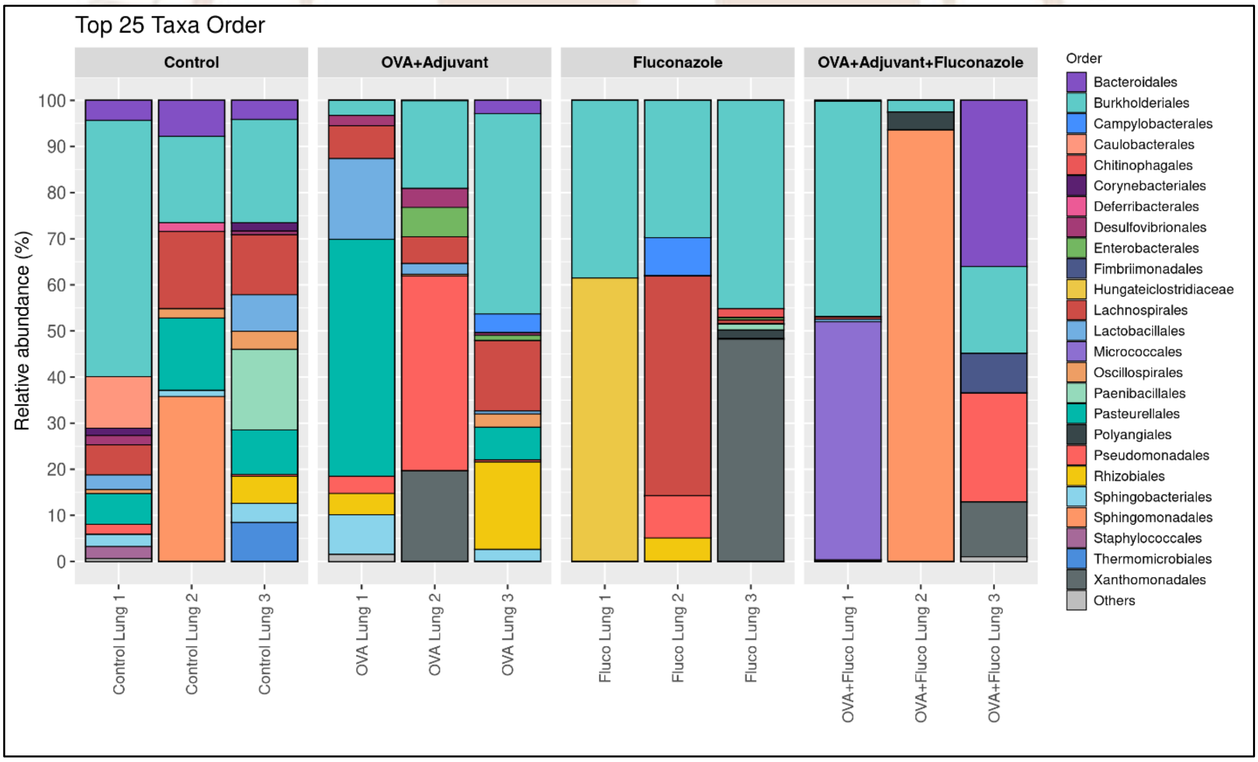
Top 25 Taxa Order
Control
OVA+Adjuvant
Fluconazole
OVA+Adjuvant+Fluconazole
Relative abundance (%)
100
90
80
70
60
50
40
30
20
10
0
Control Lung 1
Control Lung 2
Control Lung 3
OVA Lung 1
OVA Lung 2
OVA Lung 3
Fluco Lung 1
Fluco Lung 2
Fluco Lung 3
OVA+Fluco Lung 1
OVA+Fluco Lung 2
OVA+Fluco Lung 3
Order
Bacteroidales
Burkholderiales
Campylobacterales
Caulobacterales
Chitinophagales
Corynebacteriales
Deferribacterales
Desulfovibrionales
Enterobacterales
Fimbriimonadales
Hungateiclostridiaceae
Lachnospirales
Lactobacillales
Micrococcales
Oscillospirales
Paenibacillales
Pasteurellales
Polyangiales
Pseudomonadales
Rhizobiales
Sphingobacteriales
Sphingomonadales
Staphylococcales
Thermomicrobiales
Xanthomonadales
Others

Family

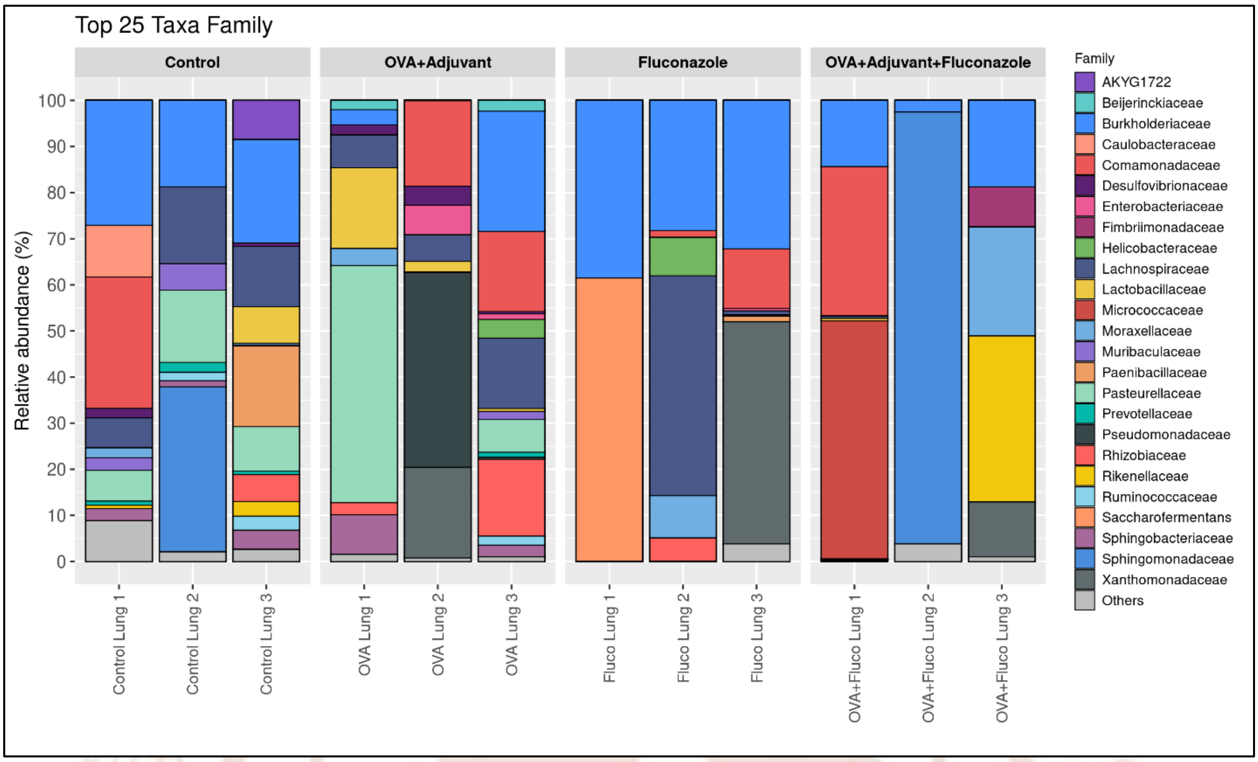

Genus

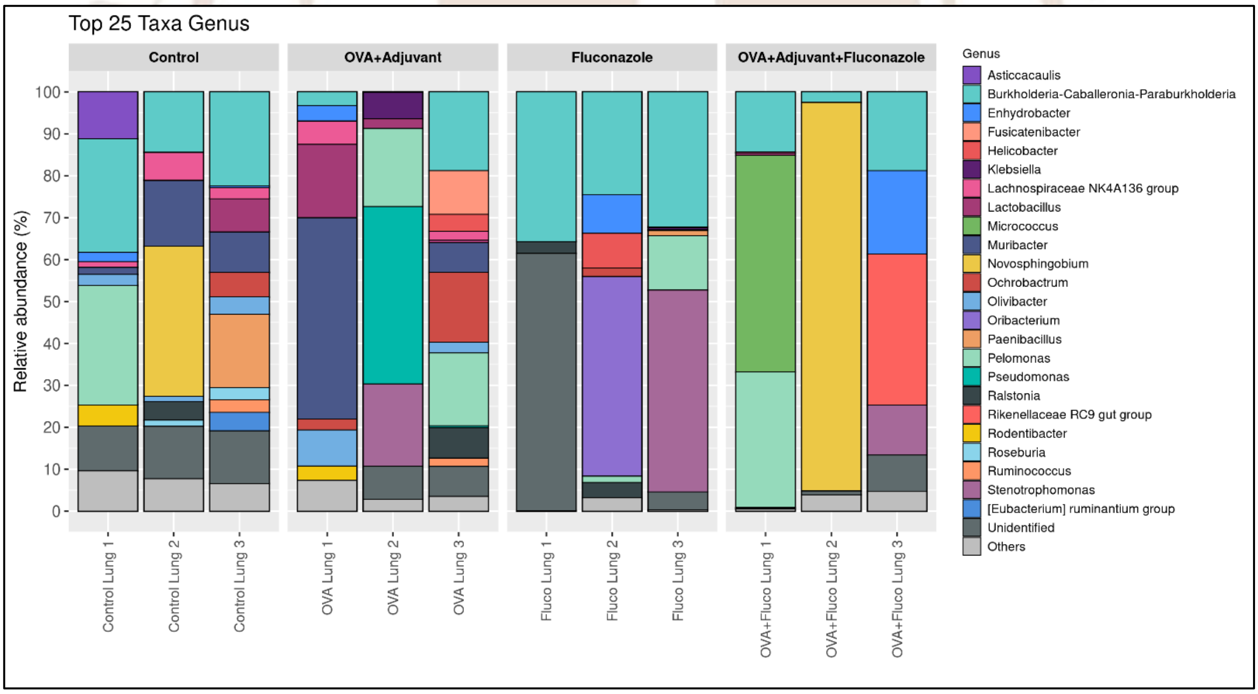

Microbial all taxa (Figure):

Phylum;	plot_bar_phylum_Group.png
	plot_bar_phylum_avg.png
Class;	plot_bar_class_Group.png
	plot_bar_class_avg.png
Order;	plot_bar_order_Group.png
	plot_bar_order_avg.png
Family;	plot_bar_family_Group.png
	plot_bar_family_avg.png

Microbial top 25 taxa (Figure):

Order;	plot_bar_order_top25_Group.png
	plot_bar_order_top25_avg.png
Family;	plot_bar_family_top25_Group.png
	plot_bar_family_top25_avg.png
Genus;	plot_bar_genus_top25_Group.png
	plot_bar_genus_top25_avg.png

Microbial all taxa (HTML):

Phylum;	plot_bar_phylum_Group.html
Class;	plot_bar_class_Group.html
Order;	plot_bar_order_Group.html
Family;	plot_bar_family_Group.html

Microbial top 25 taxa (HTML):

Order;	plot_bar_order_top25_Group.html
Family;	plot_bar_family_top25_Group.html
Genus;	plot_bar_genus_top25_Group.html

Abundance tables:

expr.abundance.all.txt
expr.relative_abundance.all.txt
expr.asv.fasta
expr.relative_abundance.phylum.txt
expr.relative_abundance.class.txt
expr.relative_abundance.order.txt
expr.relative_abundance.family.txt
expr.relative_abundance.genus.txt

Krona plot

Krona plot is multilevel pie chart used for visualizing taxonomic classification (5). The output file is an HTML file that can be accessed in the link below the figure. Figures with specific taxonomy can be captured on the HTML for publication. If your samples do not have any replicate, we can provide individual plot of Krona. In contrast, if your samples are defined into a group, the Krona plot for grouped data can be created to compare the taxa among groups.

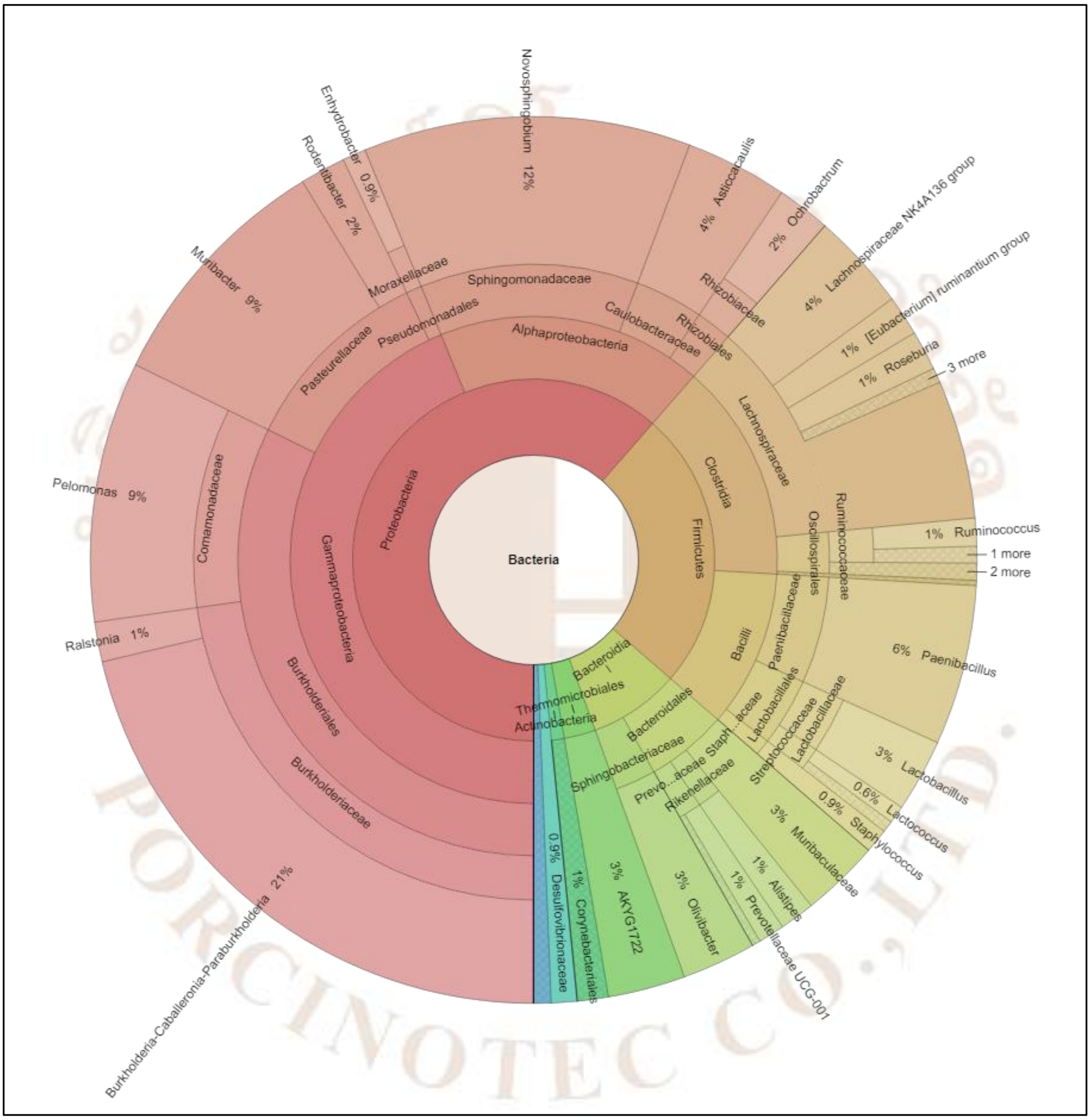

HTML:
SampleID-krona.html
Group-krona.html

6. Linear discriminant analysis effect size (LEfSe) and Cladogram

LEfSe is used to determine the significantly higher taxonomy, genes, or functions, which can explain the difference in taxa between groups (6). It is usually used to identify biomarkers between 2 or more sample groups based on bacterial relative abundances. The bar plot represents the effect size (LDA) for a significant taxon in a certain group. The length of the bar represents a log10 transformed LDA score. The colors represent which group that taxa are highly presented compared to the other group. We also provide the taxonomic table of LEfSe input as the following table.

Cladogram explains the differentially abundant taxonomic clades according to LEfSe analysis. The dot color and shading represent significantly higher abundance of taxon in a certain group. The significant phyla are presented as dots in the centre, while the significant genera are shown in the outer circle. The name of significant phyla is given in the outermost circle for colored shading. The results from both LEfSe and cladogram are similar. Please notice that the result of LEfSe shows significant difference of single taxa level, while cladogram shows the different taxonomic clades (from phylum to genus) among groups.

Suggestion by Porcinotec:

Bacterial taxa with LDA scores greater than 2 was shown in this report. Bacteria in member of *Escherichia_Shigella* (belong to phylum Proteobacteria) was the core gut microbiota in Fluconazole ($p<0.05$). The Desulfobacterota, Desulfovibrionia, Desulfovibrionales, *Desulfovibrionaceae* were highly prevalent in OVA+Adjuvant ($p<0.05$).

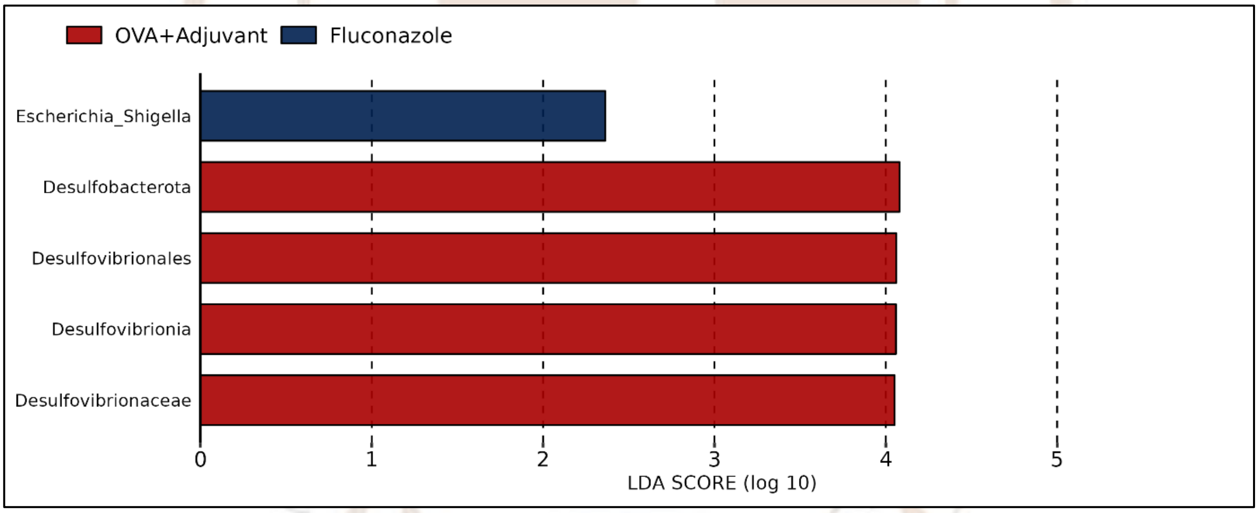

Figure:

Plot_LDA_score.png

Plot_Cladogram.png

Table:

lefse_table.txt

7. Heatmap of relative abundance

Heatmap is a graphical representation of data where different matric values are represented as different shading of colors. It is necessary for visualizing the concentration of values between two dimensions of a matrix. In this case, heatmap is used for visualizing the bacterial abundance between samples. The heatmap figure can be downloaded with the same filename below the figure.

Suggestion by Porcinotec:

Burkholderia-Caballeronia-Paraburkholderia was enrich in all samples. Eight ASVs were dominant in control, while there were rarely found in OVA+Adjuvant and Fluconazole groups. The relative abundance of *Desulfovibrionaceae*, *Muribaculaceae*, *Lachnospiraceae*, *Enhydrobacter*, *Lachnospiraceae NK4A136 group*, *Olivibacter*, *Muribacter*, and *Lactobacillus* were highly prevalent in control group. The bacterial profiles in OVA+Adjuvant were almost the same as control.

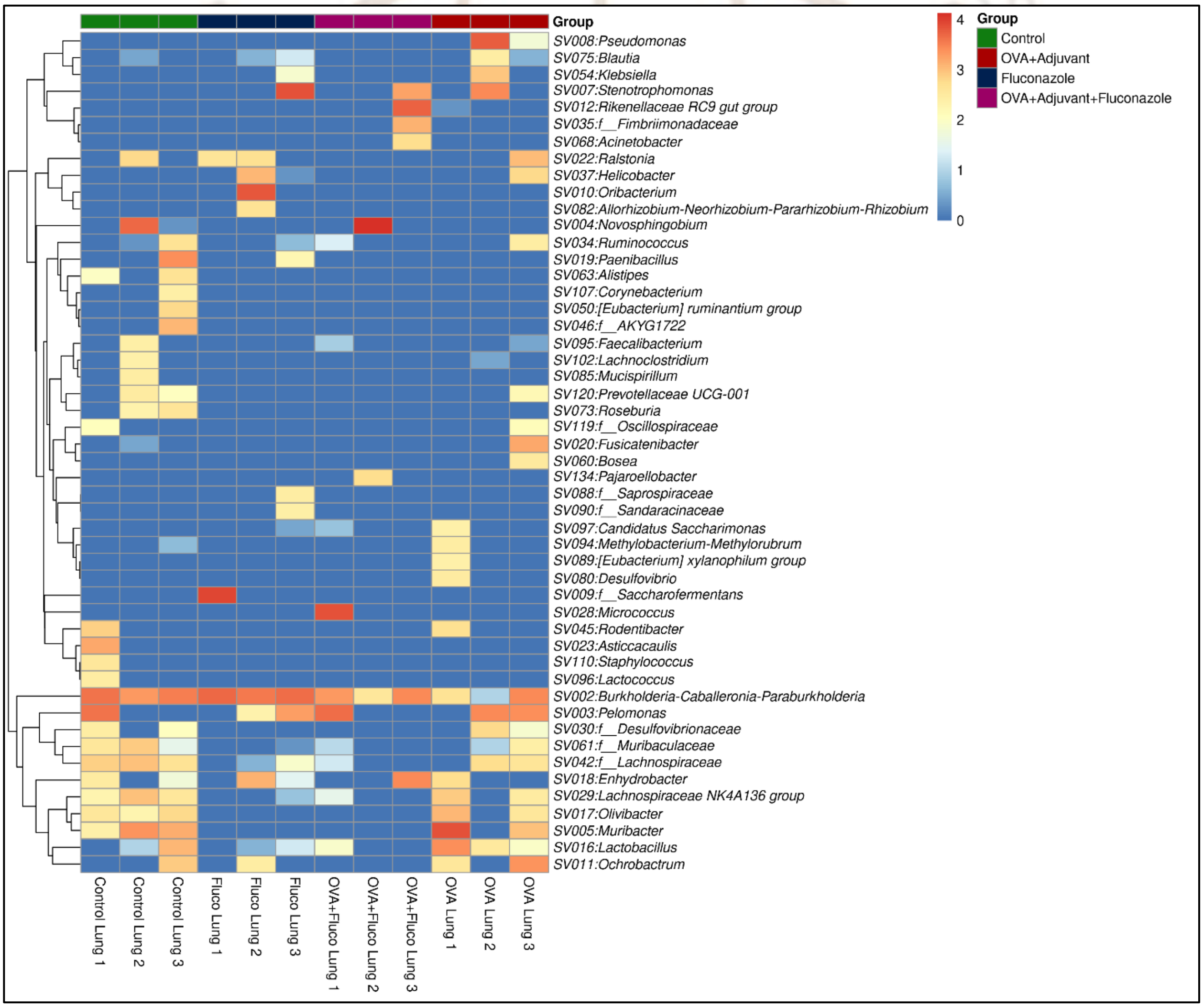

Figure:
Heat_map_label_ASV_number_top50ASVs_Genus_Group.png

8. Pipeline analysis

	Pipeline	Detail
Taxonomic reference data	Silva version 138	https://www.arb-silva.de/documentation/release-138/
Bioinformatics' pipelines	DADA2 v1.16.0	https://benjjneb.github.io/dada2/
R	R version 4.0.4 (2021-02-15)	https://www.r-project.org/
R packages	venneuler_1.1-0<br>rJava_0.9-13<br>dendextend_1.14.0<br>RColorBrewer_1.1-2<br>viridis_0.5.1<br>viridisLite_0.3.0<br>microbiomeutilities_1.00.15<br>microbiome_1.10.0<br>ggpubr_0.4.0<br>ranacapa_0.1.0<br>plotly_4.9.3<br>vegan_2.5-7<br>lattice_0.20-41<br>permute_0.9-5<br>ggrepel_0.9.1<br>ggbeeswarm_0.6.0<br>DESeq2_1.30.1<br>SummarizedExperiment_1.20.0<br>Biobase_2.50.0<br>MatrixGenerics_1.2.1<br>matrixStats_0.58.0<br>GenomicRanges_1.42.0<br>GenomeInfoDb_1.26.4<br>rexmap_1.1	forcats_0.5.1<br>stringr_1.4.0<br>dplyr_1.0.5<br>purrr_0.3.4<br>readr_1.4.0<br>tidyr_1.1.3<br>tibble_3.1.0<br>tidyverse_1.3.0<br>DECIPHER_2.16.1<br>RSQLite_2.2.5<br>Biostrings_2.58.0<br>XVector_0.30.0<br>IRanges_2.24.1<br>S4Vectors_0.28.1<br>BiocGenerics_0.36.0<br>ggplot2_3.3.3<br>phangorn_2.6.2<br>ape_5.4-1<br>phyloseq_1.34.0<br>data.table_1.14.0<br>dada2_1.16.0<br>Rcpp_1.0.6<br>microbiomeMarker_0.0.1.9000<br>pairwiseAdonis_0.0.1

PORCINOTEC CO., LTD.

9. Material and methods

16S rRNA library sequencing

The prokaryotic 16S rRNA gene at V3V4 region was performed using the Qiagen QIAseq 16S/ITS Region panel (Qiagen, Hilden, Germany). The targeted PCR was followed cycling conditions: 95 °C for 2 min, and 20 cycles of 95°C for 30 s, 50 °C for 30 s and 72 °C for 2 min, followed by 72 °C for 7 min. The 16S rRNA amplicons were purified by QIAseq magnetic beads and labeled with different sequencing adaptors using QIAseq 16S/ITS Region Panel Sample Index PCR Reaction (Qiagen, Hilden, Germany). The index PCR reaction was followed cycling conditions: 95 °C for 2 min, and 22 cycles of 95°C for 30 s, 60 °C for 30 s and 72 °C for 2 min, followed by 72 °C for 7 min. The DNA libraries with different index (approximately 630 bp) were purified using QIAseq magnetic beads (Qiagen, Hilden, Germany). The quality and quantity of DNA libraries were evaluated using DeNovix QFX Fluorometer and QIAxcel Advanced (Qiagen, Hilden, Germany), respectively. Paired-end sequencing, 2 × 300, was performed using Illumina Miseq platform following the manufacturer's protocols (Illumina, San Diego, CA, USA).

Bioinformatics analyses

The raw sequences were categorized into groups based on the 5′ barcode sequences. The sequences were processed following DADA2 v1.16.0 pipeline (https://benjjneb.github.io/dada2/). The DADA2 pipeline describes microbial diversity and community structures using unique amplicon sequence variants (ASVs) (7). Microbial taxa were classified from Silva version 138 as a reference database (8). Alpha diversity index (Chao1 richness, Shannon, and PD whole tree) was computed using DADA2 software. For Bata diversity, non-metric multidimensional scaling (NMDS) based on Bray-Curtis dissimilarity and principal coordinate analysis (PCoA) were plotted from Phyloseq data. Linear discriminant analysis effect size (LEfSe) and cladogram plot were performed to identify the bacterial biomarkers.

Data analysis

Pairwise comparison of alpha diversity (Observed ASVs, Chao1, Shannon, and PD whole tree) was calculated using Kruskal-Wallis test ($p<0.05$). Permutational multivariate analysis of variance (PERMANOVA) was performed to evaluate the significant differences for beta diversity among groups at $p<0.05$. Moreover, the Kruskal-Wallis sum-rank test was also used in LEfSe analysis to identify bacterial biomarkers that differed significantly in abundant taxon between sample groups.

Availability of Supporting Data

Nucleic acid sequences in this study were deposited in an open access Sequence Read Archive (SRA) database of NCBI, accession number SAMN37501218.

10. References

1. Willis AD. Rarefaction, Alpha Diversity, and Statistics. Front Microbiol. 2019;10:2407.

2. Jovel J, Patterson J, Wang W, Hotte N, O'Keefe S, Mitchel T, et al. Characterization of the Gut Microbiome Using 16S or Shotgun Metagenomics. Frontiers in Microbiology. 2016;7.

3. Ramette A. Multivariate analyses in microbial ecology. Fems Microbiol Ecol. 2007;62(2):142-60.

4. Xia Y, Sun J. Hypothesis Testing and Statistical Analysis of Microbiome. Genes Dis. 2017;4(3):138-48.

5. Ondov BD, Bergman NH, Phillippy AM. Krona: Interactive Metagenomic Visualization in a Web Browser. In: Nelson KE, editor. Encyclopedia of Metagenomics. New York, NY: Springer New York; 2013. p. 1-8.

6. Segata N, Izard J, Waldron L, Gevers D, Miropolsky L, Garrett WS, et al. Metagenomic biomarker discovery and explanation. Genome Biol. 2011;12(6):R60.

7. Callahan BJ, McMurdie PJ, Rosen MJ, Han AW, Johnson AJ, Holmes SP. DADA2: High-resolution sample inference from Illumina amplicon data. Nat Methods. 2016;13(7):581-3.

8. Quast C, Pruesse E, Yilmaz P, Gerken J, Schweer T, Yarza P, et al. The SILVA ribosomal RNA gene database project: improved data processing and web-based tools. Nucleic Acids Res. 2013;41(Database issue): D590-6.

PORCINOTEC CO., LTD.
